# A Cost-Driven Method for Deep-Learning-Based Hardware Trojan Detection

**DOI:** 10.3390/s23125503

**Published:** 2023-06-11

**Authors:** Chen Dong, Yinan Yao, Yi Xu, Ximeng Liu, Yan Wang, Hao Zhang, Li Xu

**Affiliations:** 1 College of Computer and Data Science, Fuzhou University, Fuzhou 350116, China; dongchen@fzu.edu.cn (C.D.); 211027013@fzu.edu.cn (Y.Y.); xuyilaser@foxmail.com (Y.X.); snbnix@gmail.com (X.L.); 2 Khoury College of Computer Sciences, Northeastern University, Boston, MA 02115, USA; wang.yan6@northeastern.edu; 3 College of Computer and Cyber Security, Fujian Normal University, Fuzhou 350007, China; xuli@fjnu.edu.cn

**Keywords:** integrated circuit security, hardware Trojan, deep learning, computational consumption, gate level, semantic analysis

## Abstract

The Cyber-Physical System and even the Metaverse will become the second space in which human beings live. While bringing convenience to human beings, it also brings many security threats. These threats may come from software or hardware. There has been a lot of research on managing malware, and there are many mature commercial products, such as antivirus software, firewalls, etc. In stark contrast, the research community on governing malicious hardware is still in its infancy. Chips are the core component of hardware, and hardware Trojans are the primary and complex security issue faced by chips. Detection of hardware Trojans is the first step for dealing with malicious circuits. Due to the limitation of the golden chip and the computational consumption, the existing traditional detection methods are not applicable to very large-scale integration. The performances of traditional machine-learning-based methods depend on the accuracy of the multi-feature representation, and most of the methods may lead to instability because of the difficulty of extracting features manually. In this paper, employing deep learning, a multiscale detection model for automatic feature extraction is proposed. The model is called MHTtext and provides two strategies to balance the accuracy and computational consumption. After selecting a strategy according to the actual situations and requirements, the MHTtext generates the corresponding path sentences from the netlist and employs TextCNN for identification. Further, it can also obtain non-repeated hardware Trojan component information to improve its stability performance. Moreover, a new evaluation metric is established to intuitively measure the model’s effectiveness and balance: the stabilization efficiency index (SEI). In the experimental results for the benchmark netlists, the average accuracy (ACC) in the TextCNN of the global strategy is as high as 99.26%, and one of its stabilization efficiency index values ranks first with a score of 71.21 in all comparison classifiers. The local strategy also achieved an excellent effect, according to the SEI. The results show that the proposed MHTtext model has high stability, flexibility, and accuracy, in general.

## 1. Introduction

A sudden coronavirus pandemic has greatly promoted the process of all humanity entering virtual life. Work from home, online learning and shopping, and the remote operation of various businesses [1], coupled with the birth of new things, such as self-driving vehicles [2], autonomous robots [3], and Industry 4.0 [4], etc., means the back-end service equipment supporting these emerging virtual businesses needs to be greatly expanded and highly relied upon. After more than two years of the epidemic, human beings have quickly entered the fourth space of life, the cyber-physical system (CPS), where human life is highly dependent on electronic devices. Hardware is the foundation of CPS, and chips are the core of hardware [5], including integrated circuits (ICs) [6], artificial intelligence (AI) chips [7], biochips [8], and so on.

Hardware has long been considered secure and trustworthy. Global research in information security is mainly focused on the software level or proposing security protocols in specific areas [9]. Malicious code [10], software vulnerabilities [11], and intrusion detection [12] are all hot research topics. Most of the security vendors are committed to solving the security threats just at the software level, and fortunately, the results in this area have been effective.

It was also discovered that some security issues come from the hardware [13], and people began to realize that threats from hardware are fatal. Since hardware is the foundation of the CPS, the security of the hardware is related to the security of the entire CPS. As human life and the CPS become more integrated, chip security issues become important [14].

Guaranteeing the security of chips is difficult. A particular chip often requires the participation of several third-party manufacturers, electronic design automation (EDA) software vendors, and intellectual property (IP) core suppliers of unknown trustworthiness. Untrustworthy implantation in any of these rings may cause the chip to deviate from its original function, such as data loss and tampering, information leakage and extortion, and functional failure and change [15].

Most ICs are made up of multiple links on a global scale, and any one of these untrustworthy links can be implanted with malicious circuits, which are called hardware Trojans (HT). Both analog and digital circuits are vulnerable to hardware Trojans [16,17].

Research teams worldwide have conducted a lot of research in developing effective hardware security countermeasures, with one of the key tasks being the creation of hardware security primitives [18]. Moreover, research into hardware Trojan detection in conjunction with ML and IP protection techniques has opened up the possibility of securing hardware [13]. As of now, it seems that malicious circuits are becoming more and more stealthy, high-hazard, and resistant to detection. Attackers are beginning to apply machine learning (especially deep learning) to side-channel attacks and reverse engineering [19,20,21]. Today, hardware Trojans are the main threat to integrated circuits (ICs) [22].

There are a lot of studies on the post-silicon stage for detecting HTs [23], such as side-channel [24], thermal map [25], and reverse engineering [26]. These post-silicon methods are indeed effective for the chips, which are not very complex or low integration. While for ultra-large-scale integrated circuits (ULSI), the existing post-silicon methods are infeasible because the malicious circuits tend to consist of very few components and account for a tiny proportion of the whole circuit. Moreover, these methods all need the golden chip as a reference, and the golden chip is hard to attain or does not exist.

For example, IBM claims its 2 nm nanosheet technology will enable 50 billion transistors to be squeezed onto a chip measuring 150 mm${}^{2}$ (about the size of a fingernail) [27]. On-chip interconnect today is based on copper/low-*k* wiring, which can be more than 100 km long [28]. The integration of the chips currently in use is very large, with diversified internal content and complex structure. In contrast, the HT circuit often occupies few parts and tends to be more hidden. Thus, activating the HT to change the overall parameters of the circuit, such as the current, power, and temperature, is insignificant. Obviously, finding the HT by observing changes in current, power, and temperature is very difficult for Very-Large-Scale Integration (VLSI).

Machine Learning (ML) is not a new subject, but today, with big data and computing energy, machine learning methods achieve excellent performance by learning from previous experiences. For finding malicious circuits on VLSI, employing Artificial Intelligence (AI) methods needs no golden chip as a reference, which is a good means of exploration. Some research teams are working on ML-based hardware Trojan detection, such as Dong et al. [29] and Kurihara et al. [30].

However, the existing methods are mainly based on traditional machine learning, which requires manual feature extraction, and the accuracy of the feature representation affects the result. The introduction of deep learning (DL) enables the automatic extraction of netlist features and results in a better generalization of the model. To address the above purpose, we proposed a DL-based approach for hardware Trojan detection to automatically extract features to identify and have a certain degree of promotion [31].

This paper proposes a cost-driven deep learning-based detection model named MHTtext. Two strategies are included in the model: a global strategy and a local strategy. The global strategy shows excellent detection performance, and the local strategy focuses more on the flexibility of the detection method to adapt to different training environments and demands dynamically. Moreover, it reduces time and economic costs. The paper also proposes a more efficient strategy fusion model to combine the advantages of both strategies.

The main contributions of this paper are as follows:

- A cost-driven deep-learning-based HT detection is proposed, which extracts the features automatically and eliminates the dependence on the comparison of the gold chip; it realizes the feasibility of detecting HTs in VLSI.
- A global–local flexible approach is proposed, which extracts the HT components from the netlist with variable length. Thus, it realizes the selectivity of the accuracy and computational consumption.
- An evaluation indicator SEI is set up and used to further measure the performance of the model. In the experiments, the stability and robustness can be shown from the SEI directly.

## 2. Threats Faced by Integrated Circuits

**Figure 1:**
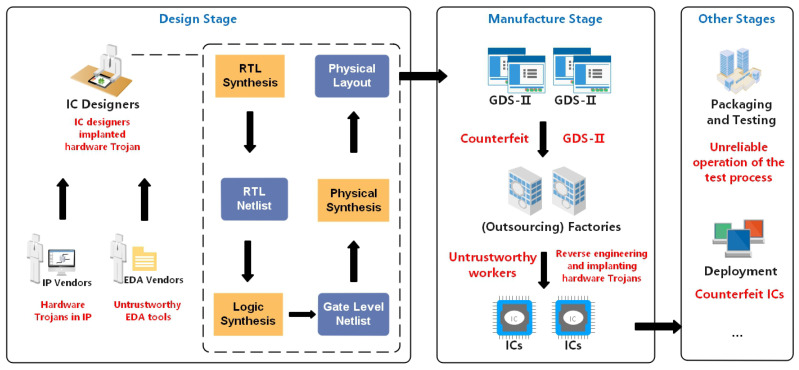
Security threats during the IC design and manufacturing process.

Figure 1 illustrates some of the security threats of the ICs from the specification design stage to the integrated packaging phase. After abstracting the production chain, it can be seen that it is mainly composed of design, manufacturing, and testing. The red parts of the figure represent the behavior or things that may lead to security threats, and the design phase tends to be more likely to reach the underlying implantation.

The design phase of ICs requires two basic vendors: the EDA vendors and the IP core vendors. The EDA tools supplied by the EDA vendors are compliant in most cases, but they may be untrustworthy when there is a security threat or malicious operator. The IP cores supplied by IP vendors can also contain malicious circuits in untrusted states [32], which are usually implanted by malicious IP designers.

When both the EDA tools and IP cores are provided intact, the IC designers start designing ICs that meet the requirements, and eventually, a GDS-II file containing the circuit logic is generated. During this process, an untrustworthy IC designer can easily implant HTs, and the untrustworthy EDA tools mentioned above also have an impact on this part of the process.

The foundry can start producing ICs after obtaining the GDS-II files. It is worth noting that the GDS-II files may be replaced by files with HTs by someone who is not trustworthy and has access to it. This results in the subsequent production of chips with HTs as well. The foundry may cooperate with several outsourcing factories [30], and these outsourcing factories or even the foundry itself may employ untrustworthy employees. The untrustworthy employees can implant HTs using reverse engineering or replace the untrustworthy ICs.

The main security threat in the packaging and testing phase comes from untrustworthy testers or test programs that allow ICs with HTs to pass the test and enter the deployment and application phase without any report. The main security threat in the deployment and application phase comes from untrustworthy deployers or implementers who replace regular ICs with HTs ICs. Then, untrustworthy ICs are thus spread throughout the field.

From the above [33], it seems that the ICs’ production chain is very simple and fragile, and the issue of hardware security has been extensively researched by academics in the recent stages. In general, there are several issues with chip design, manufacturing, and hardware Trojan detection that are still worth discussing:

**(1) The normalization of the ICs’ production chain**

In general, the chip design and manufacturing chain are intertwined, but almost every link has untrustworthy factors that may generate security threats. Therefore, complete and specific IC production specifications can better restrain the behavior of each link. Some scholars are also conducting research in this area, including the study of trustworthy behavior constraints from IP cores [34].

**(2) Hardware Trojan concealment**

Many effective hardware Trojan protection methods have made traditional hardware Trojans easily detectable in recent years. For this reason, HT implanters have also optimized their implantation methods in various ways, especially concealment. For traditional HT detection methods, to a certain extent, there is a bottleneck in the detection effect, and detection costs can not be further reduced. Thus, more and more scholars are studying the detection methods incorporated with machine learning (ML).

**(3) Limitations of the existing incorporated ML detection methods**

Most mainstream detection methods incorporating ML rely on the accuracy of multi-feature representations, and most of the methods have difficulty extracting the features manually, which may lead to the instability of the methods. In addition, reasonable control of the various costs of ML is also an issue worth discussing, and perhaps a more flexible model is needed to cope with different training environments.

## 3. Related Work

The security problem of hardware Trojans can be broadly divided into three parts: detection, localization, and failure, where the detection method of hardware Trojans has achieved excellent results by many research teams worldwide. For example, the use of a dual-mode self-test architecture approach for detecting hardware Trojans in design, manufacturing, and testing is mentioned in [35]. Sabri et al. [36] proposed an SAT-Based integrated hardware Trojan detection and a localization approach through path-delay analysis. Some related work on hardware Trojan detection will be presented in a hierarchical manner.

### 3.1. Pre-Silicon Detection

Pre-silicon hardware Trojan detection refers to the detection of HTs during the design phase, and most current low-cost detection methods are performed at this stage and can be further divided into dynamic detection techniques and static detection techniques.

#### 3.1.1. Dynamic Detection Approaches

Methods such as trust verification are used to flag suspicious circuits hidden in the netlist, and then functional or formal verification is performed to determine the presence of hardware Trojans.

Functional verification can identify some defects left in the design phase. It is simple to operate but easy to miss when the hardware Trojan has high concealment characteristics. Formal verification uses mathematical methods to verify netlists, which is a trust-based mechanism, but is also very limited when dealing with large integrated circuits. Model-checking is a common formal verification method in hardware security, and Shen et al. [37] proposed a method to speed up the verification and detection by reducing the state space, which can reduce the complexity of the model.

#### 3.1.2. Static Detection Approaches

The static detection approaches use a large amount of information extracted from the netlist, which is analyzed to determine whether it contains a hardware Trojan. This method does not require a gold chip as a reference and has the advantage of high flexibility and low cost. Many scholars have devoted a lot of time to research in this area, and based on the current literature, this part can be broadly divided into two areas.

**(1) Traditional machine learning methods**

In the field of hardware Trojan detection combined with traditional machine learning, the methods generally use shallow neural networks, random forests, SVM, and others. To a certain extent, machine learning methods solve the problems, such as the cost or environmental impact, that may exist with traditional non-static detection techniques.

Kurihara et al. [30] discussed a hardware Trojan detection method using neural networks and random forests and evaluated it using 24 benchmark tests including IP cores, which finally achieved excellent detection results. They computed 11 netlist features in their experiments.

Dong et al. [38] tried to apply XGBoost to the detection of hardware Trojans. They proposed new Trojan network features and then used XGBoost to build an effective feature set (49 out of 56 features were taken after discarding the unnecessary features according to the scoring mechanism). Excellent detection results were achieved after the final training.

ML-HTCL is a framework proposed in [29] that includes hardware Trojan detection and localization, where the detection part uses multilayer BP neural networks and SVM techniques. They used multilayer BP neural networks for the control-signal-type HTs and SVMs for the information leakage HTs.

It is worth noting that all of the above methods provide feasible solutions for hardware Trojan detection, but the methods are mainly oriented towards specific features and require some time to construct and filter the features suitable for the model. Moreover, manual feature extraction is often less convenient, and its performance is somewhat limited to feature representation. The method proposed in this paper also belongs to the static method in pre-silicon and is also applied to the gate-level netlist. For this reason, we briefly list similar solutions in Table 1.

**(2) Deep learning methods**

More and more research teams have started to use deep learning methods for static detection, which also benefits from the fact that they can extract features well automatically and achieve good results.

For deep learning, the size and quality of the dataset are two important fundamental prerequisites. Most of the existing methods use open-source data, such as the RS232-series provided by Trust-Hub, and it is worth mentioning that Liakos et al. [40] proposed a new tool, GAINESIS, a WCGAN-based algorithm that synthesizes new samples for experiments. This is a convenient tool for models where it is difficult to extract a sufficient number of samples.

### 3.2. Post-Silicon Detection

Post-silicon detection includes destructive and nondestructive detection, which is the key research area of traditional hardware Trojan detection methods.

#### 3.2.1. Destructive Detection

Destructive detection generally involves reverse engineering, which is rebuilding a new design model after a series of physical deconstruction, scanning, and analysis, then comparing it with the golden design to determine whether a hardware Trojan has been implanted. This detection method works better for early chips with simple structures, but the destructive detection makes the cost increase, and the time consumption increases accordingly. As chips become more and more integrated, it has been difficult to break the bottleneck of detection in this way. In addition, the discovery of specifications in reverse engineering is also an area for discussion [41].

#### 3.2.2. Nondestructive Detection

The main representatives of nondestructive detection are side-channel analysis and logic testing, for which more results are available in the existing literature. Yang et al. [42] proposed a multidimensional self-referencing technique that does not require a gold chip, based on the traditional side-channel analysis method. With a fully automated detection framework, it can reliably detect minor hardware Trojans. The side-channel-dependent approach proposed by Karabacak et al. [43] was also based on self-referencing, and did not rely on plausible samples. Zhu et al. [44] proposed a Jintide architecture to verify the chip at runtime using a trusted external monitor. Chen et al. [45] proposed a holistic self-testing approach to directly detect security threats and malicious attacks on sensors. Taheri et al. [46] proposed an efficient integrated HT detection technique based on the evaluation of integrated parasitic capacitance variations, which did not require the support of a gold IC and could use simulated data to detect HT. Wen et al. [47] proposed a nondestructive method based on thermal maps and inception neural networks, where thermal maps of various sample IC chips were collected and then optimized, and the optimized data were analyzed using INN. The experiments were also performed with the help of custom filters and achieved better results.

### 3.3. Detection and Prevention for Specific Chips

There are many research teams working on certain specific types of chips.

Alhelaly et al. [48] proposed a method for detecting hardware Trojans in 3D integrated circuits and also explored the performance of this detection method when the attacker modified the characteristics of TSVs. Cho et al. [49] proposed a bidirectional mechanism to detect hardware Trojans in FPGAs at any stage in order to efficiently detect them. Similarly, for FPGA research, Ma et al. [50] proposed an on-chip security framework that can be used to verify whether an HT has tampered with or corrupted the original design. Mohd et al. [51] implemented a run-time monitoring design for lightweight ciphers in RCDs on an FPGA platform. Hossain et al. [35] proposed a safety model to avoid IP theft from microfluidic biochips at various stages of the biochip design process for drive sequences. Moreover, the joint extension of split manufacturing and camouflaging techniques to 3D integration in [52] is an important step forward in the prevention of hardware Trojans.

The remainder of this paper is organized as follows: Section 2 gives the description of HT treats of chips. Section 3 lists the related work of HT detection. Section 4 describes the HT detection problem with formulas. Section 5 shows the dataset generation of the netlists, including path sentences’ generation with the two strategies. Section 6 mainly describes the deep learning architecture. Section 7 shows the experiment details and analyzes the performance of the MHTtext. Section 8 gives the discussion about the MHTtext. Finally, Section 9 provides the conclusion.

## 4. Problem Formulation

In this section, the problem formulation of the MHTtext model is introduced, including the mathematical abstraction of global/local-features-based detection models and the goals of the paper.

### 4.1. Problem Description

Hardware Trojans are implanted in circuits at the design stage more often than not; thus, the paper proposes the MHTtext model, which is a TextCNN-based pre-silicon detection for hardware Trojans. The attack pattern of the design stage defined in the paper is shown in Situation 1.

Situation1 Consider a series of chip netlists $R=\left\{r_{1},\;r_{2},\;\ldots ,\;r_{p}\right\}$; there could be a set of HT components, which is implanted into the chips, and it is denoted by $H=\left\{h_{1},\;h_{2},\;\ldots ,\;h_{m}\right\}$. When the HTs are not triggered in response, the HTs can coexist with the normal circuit and do not affect the function of the chip. Once the rare conditions $C=\left\{c_{1},\;c_{2},\;\ldots ,\;c_{k}\right\}$ or signals $S=\left\{s_{1},\;s_{2},\;\ldots ,\;s_{v}\right\}$ are released by the triggers, the payload is activated, and the paths $P=\left\{p_{1},\;p_{2},\;\ldots ,\;p_{q}\right\}$ containing HTs will perform abnormal behaviors, which cause damage such as signal tampering through signal transmission. *p*, *m*, *k*, *v*, and *q* mean the number of netlists, components, conditions, signals, and paths, respectively.

For TextCNN to be effective in detecting the HT netlist at model runtime, a full description of the problem is shown as follows:

- ***Input:*** **(i)** The chip netlist files, which consist of *l* components, denoted by $N=\left\{n_{1},\;n_{2},\;\ldots ,\;n_{l}\right\}$, and *t* wires, denoted by $W=\left\{w_{1},\;w_{2},\;\ldots ,\;w_{t}\right\}$; **(ii)** The multiscale-based algorithm that can search the specific paths including the simple path $f_{spath}:w_{i}\to w_{j}$ from the input port wire $w_{i}$ to the output port wire $w_{j}$ and the local path $f_{lpath}:n_{e}\to n_{e+2\delta }$ from the start component to the end component, where δ represents the predefined search scope.
- ***Output:*** A detection result for judging whether the path sentence $T_{spath}/T_{lpath}$ is an HT path sentence $T_{spathHT}$/$T_{lpathHT}$ (transformed from HT path $f_{spathHT}/f_{lpathHT}$).
- ***Objective:*** Maximize SEI ($SE_{HT}$ and $SE_{overall}$).

Table 2 presents the symbols and notations used throughout this paper; some that are easy to understand in a specific context are not shown here.

### 4.2. MultiScale Framework Description

The scale of the netlist, which is used to describe the connections between circuit components, is not constant. When there is a large-scale netlist in practical application, an algorithm could perform ordinarily in terms of time efficiency, though it performs well in terms of accuracy in the final recognition task. The global strategy proposed in this paper has this property. For this reason, the model is shaped anew, and a strategy based on a local topology is proposed, which significantly reduces the time overhead. In addition, it is worth discussing how to rationalize the algorithms used. In this part, a possible subjectively controlled approach is presented and attempts to solve the problem.

In order to build a subjectively controlled framework, we incorporate global/local strategies into preprocess integrated operations with a threshold θ for subjective input, where θ means the unacceptable length of the logic component definition part mentioned in Definition 3 (signal flow logic). Consider an HT-contained netlist dataset, which is recognized as a reasonable scale of the past work to be used as the standard, such as RS232-T1000.

(1) θ=Θ,

where Θ represents the length of the logic component definition part with a preset standard netlist dataset such as RS232-T1000.

More subjectively, we set a default value $\theta =\theta^{\prime }$ for the subjective input based on previous experience.

After the threshold θ is determined, each netlist file that enters the preprocessing session will be classified with different strategies selected based on simple scale perception. Further, a weighted approach with score items would make this threshold closer to the true acceptability of different projects.

Assuming that the effect of the current model is only influenced by the global and local strategies, it can be represented as follows:

(2) $$E\left(\theta \right)=E_{loc}\left(\theta \right)+E_{glo}\left(\theta \right),$$

where E(θ), $E_{loc}\left(\theta \right)$, and $E_{glo}\left(\theta \right)$ are the total effect, the effect of the local strategy, and the effect of the global strategy, respectively. Moreover, $E_{loc}\left(\theta \right)$ and $E_{glo}\left(\theta \right)$ are made up of the costs and benefits of the two strategies respectively as follows:

(3) $$E_{loc}\left(\theta \right)=\sum_{\tau =1}^{T}\sum_{i=1}^{n}H\left(L_{i}^{\tau }-\theta \right)\left(-\alpha^{\tau }EF_{i}^{\tau }+\left(1-\alpha^{\tau }\right)A_{i}^{\tau }\right)$$

(4) $$E_{glo}\left(\theta \right)=\sum_{\tau =1}^{T}\sum_{i=1}^{n}H\left(-L_{i}^{\tau }+\theta \right)\left(-\alpha^{\tau }EF_{i}^{\tau }+\left(1-\alpha^{\tau }\right)A_{i}^{\tau }\right),$$

where *n* is the total number of netlists, *i* is the current netlist, and τ represents a specific period of time for the model to run. *L* and θ denote the length of the netlist and the set unacceptable value, respectively. α is the experimentally set preference value, which is used to express the focus between the costs and benefits. EF and *A* denote the costs and benefits, respectively, which are generally expected to be minimized and maximized. These abstract models are validated in the subsequent experimental section with extreme cases, where a simple description is desired to demonstrate the detection problem. H(x) denotes the Heaviside function, which is intended to be used here to achieve a preference for the use of global and local strategies and can be expressed as follows:

(5) $$H\left(x\right)=\left\{\begin{array}{cc} 1, & x\geq 0,\\ 0, & x<0. \end{array}\right.$$

To further explain $EF_{i}^{\tau }$, where it consists of a time cost and a space cost:

(6) $$EF_{i}^{\tau }=T_{i}^{\tau }+S_{i}^{\tau }.$$

Here, the costs and benefits are normalized so that the values can be mapped to between 0 and 1:

(7) $$T_{i}^{\tau }=\frac{t_{i}^{\tau }-t_{min}^{\tau }}{t_{max}^{\tau }-t_{min}^{\tau }}$$

(8) $$S_{i}^{\tau }=\frac{s_{i}^{\tau }-s_{min}^{\tau }}{s_{max}^{\tau }-s_{min}^{\tau }}.$$

Again normalizing for $A_{i}^{\tau }$:

(9) $$A_{i}^{\tau }=\frac{a_{i}^{\tau }-a_{min}^{\tau }}{a_{max}^{\tau }-a_{min}^{\tau }}.$$

With Formulas (1)–(9) above, the objective of the framework also becomes clear, and the optimal θ can be expressed as:

(10) $$\theta_{opt}=argminE\left(\theta \right).$$

### 4.3. The Overall Flow of MHTtext

**Figure 2:**
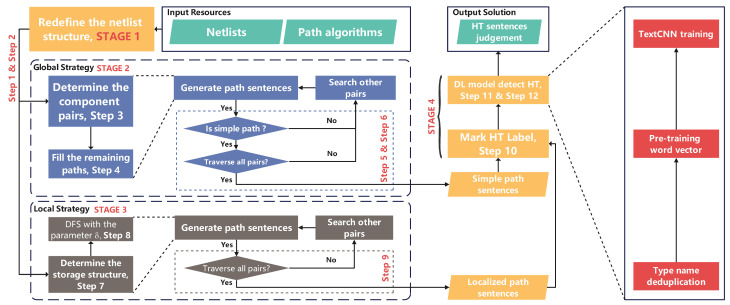
Overall flow of MHTtext.

To solve the abstract problem raised above, this paper proposes the MHTtext model as shown in Figure 2. We redefine the netlist structure through the netlist code to extract the features of the components and wires. The contents of the netlist structure are divided into three categories and further reshaped in accordance with global/local-features-based algorithms. Furthermore, the algorithms generate specific sentences to fit two search patterns. To judge the extra HT path, the generated path sentences are labeled with HT and trained through word vector pretraining and TextCNN deep learning. The proposed architecture is the MHTtext model that can solve the above HT netlist detecting problem. The detailed step-by-step description is shown below:

**STAGE I: Netlist preprocessing**

**Step 1.**  Redefine the input netlist in the way needed for the two strategies, including the three parts: the port definition, the wire definition, and the logic component definition;
**Step 2.**  Select the strategies according to the predefined patterns; the global strategy will go to STAGE II, and the local strategy will go to STAGE III;

**STAGE II: Global strategy**

**Step 3.**  Determine the start component node and the end component node and combine the two components to obtain the component pair;
**Step 4.**  Use the component pairs as input to the path-filling algorithm to generate path sentences;
**Step 5.**  Determine whether the generated path sentence is a simple path, and if so, proceed to Step 4, otherwise search for other component pairs;
**Step 6.**  Determine whether the traversal of all component pairs are complete. If so, generate the set of simple path sentences, otherwise search for other component pairs;

**STAGE III: Local strategy**

**Step 7.**  Transform the redefined netlist to an adjacency list. It is also possible to use the adjacency matrix to easily calculate some information about opposite paths;
**Step 8.**  Determine the parameter δ and then DFS (Depth First Search) the netlist to generate local path sentences;
**Step 9.**  Determine whether the traversal of all component pairs is complete. If so, generate the set of simple path sentences, otherwise search for other component pairs;

**STAGE IV: ML model**

**Step 10.**  Label the generated sentences and input them into the DL model, which will be used for the determination of HT sentences after the model is trained;
**Step 11.**  Select the type names from the utterances and discard other useless data. The type of the components should be more appropriate to express the feature;
**Step 12.**  Perform pretraining of the word vectors and the training of TextCNN. This step usually requires several parameter adjustments to obtain the best performance of the model.

## 5. Dataset Generation

In this section, the netlist preprocessing and sentence generating are introduced. The former is responsible for transforming the netlist file into a TextCNN-fit dataset, and the latter is the core link in the transformation process.

### 5.1. Redefinition for Netlist Structure

The netlist is a file used in circuit design to describe the connections between the circuit components. In this paper, a gate-level netlist is used, i.e., the circuit elements are the gate-level or the same level as them. In order to sort out the information needed to generate sentences, the structure of the netlist needs to be rationalized.

**Figure 3:**
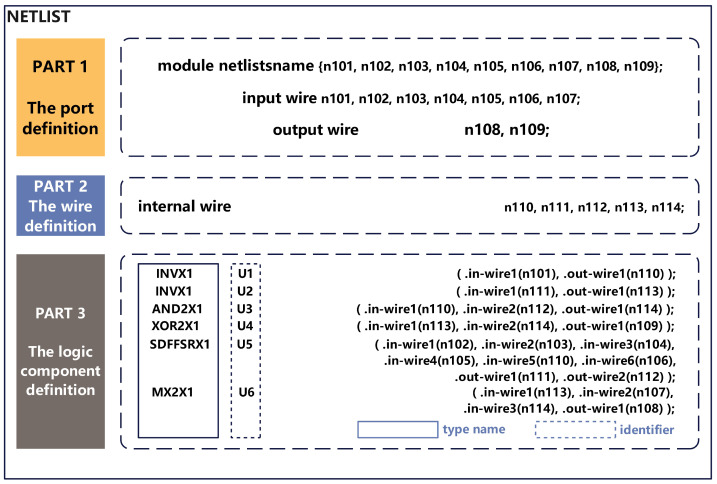
Netlist code structure division.

Figure 3 shows the netlist file written in Verilog language. The contents of the code segments are divided into three categories: the port definition, the wire definition, and the logic component definition.

**Definition** **1.** *The port definition part: This part consists of the module netlist names, the input wires, and the output wires. The wires connected to the input or output ports and the mathematical representation can be shown as:*

(11) $$M=W_{input}\cup W_{output},$$

*where M, $W_{input}$, and $W_{output}$ mean the set of module netlist names, input wires, and output wires, respectively.*

**Figure 4:**
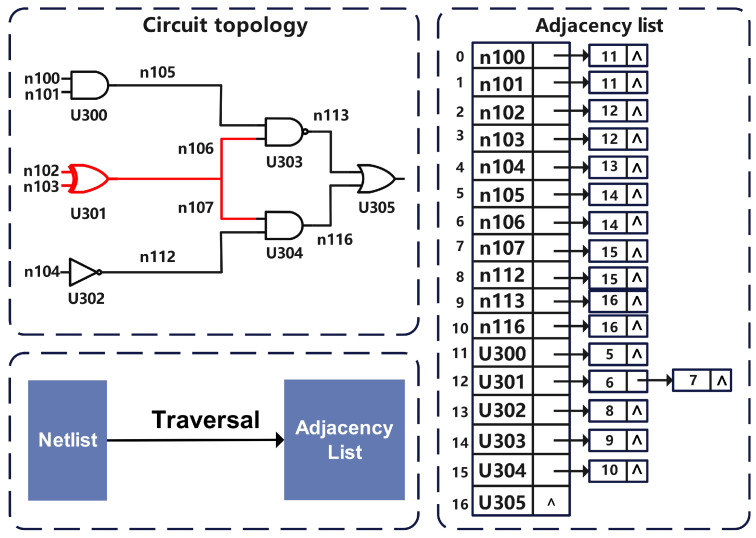
Storage structure for generating sentences.

**Definition** **2.** *The wire definition part: This part lists the internal wires in the circuit and their identifier information. Different from the wires in Definition 1, the internal wire is the wire connecting the circuit elements to each other. For example, in Figure 4, n102 connects circuit element U301 to the input port, so it is considered an input wire; n112 connects two circuit elements (U302 and U304), so it is considered an internal wire. Figure 3 and Figure 4 represent two separate netlists.*

(12) $$W_{internal}\cap \left(W_{input}\cup W_{output}\right)=\emptyset ,$$

*where $W_{internal}$ and W mean the internal wires and the wire set mentioned in Section 4, respectively, and $W_{internal}\subseteq W$.*

**Definition** **3.** *The logic component definition part: This part shows a complete circuit logic. In each row, it contains the only component with its type name, identifier, and in-wire/out-wire information. The signal from the in-wire is sent to each component, which outputs the signal via the out-wire after a logic operation is processed.*

We iterate through each row of the logic component definition part defined in Definition 3; the diagram of the circuit structure can be given, and the signals transmitted between adjacent components follow the principle of that for the wires with the same identifier. Figure 4 shows the storage structure of the generated sentences.

### 5.2. Definition of the Circuit Signal Transmission Law

The expression of word order in generated sentences is related to the way of the signal is transmitted. The detection model relies on the following law to form a recognizable text dataset.

The components set $N=\left\{n_{1},\;n_{2},\;\ldots ,\;n_{l}\right\}$, mentioned in Section 4 is the only identifier provided by *the logic component definition part* that corresponds to the specific component $n_{h}\;\left(n_{h}\in N\right)$. The wire set $W=\left\{w_{1},\;w_{2},\;\ldots ,\;w_{t}\right\}$ does too. We consider the components $n_{i}$, $n_{j}\;(1\leq i,\;\leq l$, 1≤j≤l, i≠j, $n_{i}\in N$, $n_{j}\in N)$ and introduce tuples $\xi_{i}$, $\xi_{j}$ to record each specific component and its in-wire/out-wire information, respectively.

(13a) $$\xi_{i}=\left(n_{i},\;X_{ii},\;X_{io}\right)$$

(13b) $$\xi_{j}=\left(n_{j},\;X_{ji},\;X_{jo}\right)$$

(13c) $$\xi =\left\{\xi_{1},\;\xi_{2},\;\ldots ,\;\xi_{l}\right\}.$$

The sets of the in-wire and out-wire of component $n_{i}$ are represented as $X_{ii}$ and $X_{io}$, respectively, and $X_{ji}$ and $X_{jo}$ also mean the sets of the in-wire and out-wire of $n_{j}$ ($X_{ii},\;X_{io},\;X_{ji},\;X_{jo}\subset W$), respectively.

With the above predefined context, the description of the signal transmission law can be given:

(14) $$X_{io}\cap X_{ji}\neq \emptyset \Rightarrow f_{s}:n_{i}\to n_{j},$$

where $f_{s}$ is the intuitive representation of the signal transmission, and the binary operation section is the core condition that describes the transmission of signals between components, which means the circuit signals can transmit from $n_{i}$ to $n_{j}$, since they have the same identifier in the out-wire and in-wire sets, respectively.

### 5.3. Global Strategy for Dataset Generation

The global-view-based sentence generation is the basic strategy of the MHTtext model. Its common realization pattern keeps the model from being complicated. A brief description of the global strategy can be found in [31]. This section shows the details of the global strategy and proposes a new local strategy and balancing scheme.

#### 5.3.1. Simple Path Sentence Generation

To gain the text materials formed from simple path sentences, the algorithm processes the netlist through semantic understanding. TextCNN model learns these simple path sentences to understand the circuit layout and recognize the extra HTs after complete training.

Based on the global strategy, the netlist features’ extracted framework and simple path sentences’ generation function are introduced as follows:

**(1) Determine the start/end component**

We consider a global strategy, in which a complete signal transmission path from the start node to the end node needs to be converted into TextCNN-fit text data; thus, the first step is to determine the port identifier and recognize the start component and the end component on a path pair.

To accurately represent this work, a common situation is defined for subsequent identification.

Situation2 The input port wire set and output port wire set are analyzed from the netlist, denoted by $X_{iport}$ and $X_{oport}$ ($X_{iport},\;X_{oport}\subset W$), respectively. For the specific component $n_{i}$, the sets of in-wire and out-wire are represented as $X_{ii}$ and $X_{io}$, respectively, mentioned in Section 5.2.

Therefore, the work of identifying the start and end components can be defined with a binary operation as:

(15) $$X_{ii}\cap X_{iport}\neq \emptyset \Rightarrow n_{i}\in SC$$

(16) $$X_{io}\cap X_{oport}\neq \emptyset \Rightarrow n_{i}\in EC,$$

where SC and EC represent the node sets of the start components and end components, respectively. The binary operation section is the core condition in judging. Once finding the same identifier within the in-wire set and in-port set, the model can believe there is one of the start components in the circuit. Formula (15) shows how component $n_{i}$ is recognized as a start component, for example. In Formula (16), we use a similar approach to find one of the end components in the circuit.

After finding the start component and the end component, the model considers them as the first and the last words for a specific path sentence. Assuming a path sentence contains these words, it is called a global-view path sentence, which can completely reflect a panoramic view of the signal transmission throughout the circuit.

**(2) Path searching and simple path judging**

Determining the start component and the end component is the first step of the global strategy. After the first and last words are recognized, the model can further complement the remaining components of the complete path sentence. In most cases, it can be obtained according to the proposed formula:

(17) $$Path\left(n_{w},\;n_{v}\right):f_{s}^{\varepsilon }\left(n_{w}\right)=n_{v}$$

(18) $$Path\left(n_{w},\;n_{v}\right)=\left\{n_{w},\;n_{t1},\;n_{t2},\;\ldots ,\;n_{\varepsilon -1},\;n_{v}\right\},$$

where $n_{w}$ is the start component, $n_{v}$ is the end component ($n_{w}\neq n_{v}$), and ε represents the number of iterations of the function $f_{s}$ mentioned in Formula (14). Each iteration starts from the current component to find the next that matches the signal transmission law, and after ε iterations, we finally find one of the end components $n_{w}$.

The generated path $Path\;(n_{p},\;n_{q})$ consists of a collection of components. However, when the algorithm searches without certain rules when performing iterations, it generates a large number of paths with a lot of invalid nodes. The next work of the model is therefore to find simple paths that meet the requirements according to certain rules.

The following formula expresses promising constraints for generating a simple path:

(19) $$SimplePath\left(n_{p},\;n_{q}\right):f_{sp}^{\varepsilon }\left(n_{p}\right)=n_{q}$$

(20) $$SimplePath\left(n_{p},\;n_{q}\right)=\left\{n_{p},\;n_{e1},\;n_{e2},\;\ldots ,\;n_{\varepsilon -1},\;n_{q}\right\}$$

(21) $$\forall \alpha ,\;\beta \left(f_{s}^{\alpha }\left(n_{p}\right)\neq f_{s}^{\beta }\left(n_{p}\right)\right)\Rightarrow f_{sp}^{\varepsilon }\left(n_{p}\right),$$

where 0≤α≤ε,0≤β≤ε,α≠β. $f_{s}^{\alpha }\left(n_{p}\right)$ and $f_{s}^{\beta }\left(n_{p}\right)$ are any two unequal components that are populated for the specific path during the iteration. The function for generating a simple path is called $f_{sp}^{\varepsilon }$.

With Formula (21), the model generates the simple path $SimplePath(n_{p},\;n_{q})$. It consists of a collection of nonrepetitive components, i.e., each component element in the search path (corresponding to the word in the path sentence) is different from any other element in the path, subject to the constraints of Formula (21).

**Figure 5:**
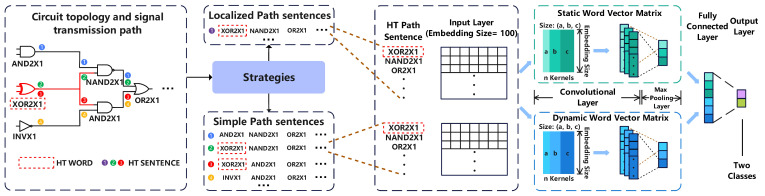
The sample distribution in the comparison models.

Based on Formulas (17) and (21), some simple paths extracted from the netlist can be identified conclusively. The left part of Figure 5 shows a simplified example. For each simple path, the identifier of a specific component is recorded as a word in the generated sentence when a signal is transmitted to it in the path. The circuit components drawn in red in the diagram are HT components. Therefore, an HT path has been identified, and a sentence has been generated for such a netlist called the HT sentence.

#### 5.3.2. The Global Strategy Algorithm Description

In many cases, loop features are also extractable information, but the method proposed in this section uses non-loop features. In previous studies [29,38], loop features have not achieved a good evaluation advantage for detecting HTs. In [38], although the loop features played a significant role in hardware Trojans’ detection work, the proportion of these particular loop features was relatively low. The method proposed in this section shows that in further experiments, better detection results can be achieved using only simple paths; they demonstrate the feasibility of this attempt to some extent.

### 5.4. Local Strategy for Datasets’ Generation

In this section, a sentence generation algorithm based on a local path strategy is presented. The freely controllable parameter δ in the algorithm allows the algorithm to be more flexible when discovering local paths.

#### 5.4.1. Local Path Sentence Generation

With the same aim as the previous strategy, the algorithm processes the netlist through semantic understanding. The TextCNN model learns local path sentences to understand the local circuit layout and recognizes the extra HTs after a complete training.

Based on the local strategy, the netlist features’ extracted framework and local path sentences generation function are introduced as follows:

**(1) Netlist storage structure and sentence generation format**

The algorithm proposed in this part is based on Depth First Search (DFS) and introduces a custom search scope δ to extract local features from the netlist.

To facilitate the search that follows, the wires and components in the gate-level netlist are treated here as a node in the graph structure, and the connected nodes (wires or components) of each node are then stored as an adjacency list. After processing is complete, each node in the adjacency list has a unique identifier (i.e., the identifiers mentioned in Section 5.1). Figure 4 shows the schematic diagram of the storage structure.

The next step is to find a suitable sentence format to reflect the local characteristics of the circuit. The paper gives a meaningful sentence format, which is defined as follows:

**Definition** **4.** *The unidirectional multiparameter format: Consider a specific path* $P=\left\{n_{1},\;n_{2},\;\ldots ,\;n_{u},\;\ldots ,\;n_{f}\right\}$*, where there exist parameters η, μ that satisfy:*

(22) η=u−1

(23) μ=f−1

(24) $$MPath=(P_{\eta },\;P_{\mu }),$$

*where $P_{\eta }=\left\{n_{1},\;n_{2},\;\ldots ,\;n_{u}\right\}$, $P_{\mu }=\left\{n_{1},\;n_{2},\;\ldots ,\;n_{f}\right\}$, η and μ represent the search scopes in sample i, and $n_{1}$ is a specific component in P on which a multisearch is executed based on $n_{1}$, generating a path tuple $Mpath=(P_{\eta },\;P_{\mu })$. With the Formulas (22)–(24), a sentence can be transferred from tuple Mpath, and it is called a unidirectional multiparameter format sentence.*

For the unidirectional multiparameter format, the time required to process the extracted sentences has been reduced, but longer sentences still need to be generated to achieve higher recognition rates. Thus, the paper considers a sentence format that takes into account the before-and-after effects of a specific component:

**Definition** **5.** *The bidirectional single-parameter format: Consider a specific synthesis path* $P=\left\{n_{1}^{-},\;\ldots ,\;n_{u}^{\pm },\;\ldots ,\;n_{f}^{+}\right\}$*, where there exists a parameter δ that satisfies:*

(25) δ=u−1=f−u,

*where δ represents the search scope,* $n_{f}$ *is a specific end component in* P*, + denotes a forward topology sequence, − denotes a reverse topology sequence, and* $n_{u}^{\pm }$ *is the common node. With Formula (25), the path* P *is called the bidirectional single-parameter path and its central node is* $n_{u}^{\pm }$*. The sentence transferred from* P *is considered a bidirectional single-parameter format sentence.*

**(2) Path searching and sentence extraction**

Determining the format of the sentences is the first step of the local strategy. Once the netlist data storage structure has been defined, the DFS can be started.

Depth First Search is a kind of graph algorithm; the process, in brief, is to move as deep as possible into each existing branching path, and each node can only be visited once. That is, when all the edges of a node *n* have been explored, the searching goes back to the start node, where the edge of the node *n* is found. This process continues until all the nodes are reachable from the source node. If there are still undiscovered nodes, one of them is selected as the next source node, and the process is repeated. The process is repeated until all nodes have been visited.

The path obtained by traversing the circuit in the standard signal flow direction is called the forward path. On the contrary, the path obtained by traversing the circuit after processing the signal flow direction is called the reverse path.

After the depth priority sequences are given as topological sequences: $P^{+}=\left\{n_{1},\;n_{2},\;\ldots ,\;n_{u},\;\ldots ,\;n_{f}\right\}$ and reverse topological sequences: $P^{-}=\left\{p_{1},\;p_{2},\;\ldots ,\;p_{u},\;\ldots ,\;p_{f}\right\}$, the local path can be obtained from:

(26) $$\exists q_{i}\in \left(P^{+}\cap P^{-}\right)\left(i\geq \delta +1\;\;\;and\;\;\;i\leq f-\delta \right)\Rightarrow f_{lp}\left(n_{i}\right)$$

(27) $$LocalPath\left(n_{i}\right)=\left\{q_{i-\delta }^{-},\;\ldots ,\;q_{i},\;\ldots ,\;q_{i+\delta }^{+}\right\},$$

where δ is the search scope, and $LocalPath(n_{i})$ is considered a bidirectional single-parameter format path. Moreover, the front and back of $q_{i}$ are forward and reverse topological sequences, respectively; + denotes forward, and − denotes reverse. Note that $q_{i}$ is a common node of $P^{+}$ and $P^{-}$. Unlike the previous strategy, here, sentence generation relies on the component words that pass through the first few levels of the central word in the signaling direction and the component words that pass through the next few same levels in the reverse signaling direction to combine into sentences. On the one hand, this is an effective way to explore the “central word” as the core path feature, and on the other hand, it is also different from the cut of paths (subpaths of end-to-end paths) generated in the global strategy. Keeping the same search scope in the forward and reverse directions can control the overall path length and simplify the problem.

The reverse topology sequence is preserved by the stack introduced during the search, and the parameter δ is not an arbitrary value. Considering the rate of local features the model needs, this parameter should be set with care. In the experimental section, several sets of data obtained through testing are presented to initially determine the value in this paper. δ should reflect different meanings in different environments and needs. In addition, if the parameter δ is set sufficiently large, the algorithm degenerates to a DFS search of the graph in the global field of view.

#### 5.4.2. The Local Strategy Algorithm Description

The proposed Formulas (26) and (27) generate several local paths the algorithm needs. After the local paths are determined, the sentences are transformed from them and used for the next training. The local-strategy-based text preprocessing algorithm is introduced in Algorithm 1 in detail.

The algorithm takes a gate-level netlist *L* and a custom search range parameter δ as input and ultimately outputs locally characterized sentences. First, the algorithm abstracts all three parts of the netlist content to the adjacency list to obtain AL. Then, it performs a DFS search on AL to generate a depth first search sequence P. Finally, all elements in P are traversed to find the sentences that match the strategy.

The condition in line 8 of the algorithm is defined in this part as:

Condition1 *(Length of LocalPath)* In this part, the max length of LocalPath is set to 300,000. In fact, it needs to be adapted to the specific situation. The 300,000 is set based on the requirements of the designed training model.

Eventually, the generated local sentences would be imported into the TextCNN model for training.
**Algorithm 1** The Local Strategy Algorithm**Input:** the specific netlist *L* and the search scope δ**Output:** the local path sentences  1:Iterate through *L* to generate the adjacency list AL;  2:$P^{\pm }=DFS\left(AL\right)$  3:**for** $q_{i}$ in $\left(P^{+}\cap P^{-}\right)$ **do**  4:      **if** i≥δ+1andi≤f−δ **then**  5:           $LocalPath\left(n_{i}\right)=\left\{q_{i-\delta }^{-},\;\ldots ,\;q_{i},\;\ldots ,\;q_{i+\delta }^{+}\right\}$;  6:           $LocalPath(n_{i})\in LocalPath$  7:      **end if**  8:      **if** meet the condition **then**  9:           continue;10:      **end if**11:**end for**12:**for** item in LocalPath **do**13:      LocalPathSentence← Extract the type name of each component in item;14:**end for**15:**return** LocalPathSentence.

## 6. Pretraining and Deep Learning Architecture

In this section, the details of pretraining and the model structure of TextCNN are introduced. Moreover, some parameters in the model are given here.

### 6.1. Type Names Pretraining

In this part, the work on type name pretraining is presented. In the work mentioned above, the MHTtext model extracted the type names from the generated sentences. The pretraining of type names lets the word vectors record the relative relationships of the circuit components at that location. To better reflect the correlation between words in the word vector, we use unsupervised learning, word2vec, to calculate the clustering relationships between all words in the vocabulary after de-duplication.

#### 6.1.1. Selection of Word Vectorization Pattern

The common approaches for word vectorization are one-hot encoding, the TF-IDF model, and the Word2vec model. One-hot coding and the TF-IDF model are bag-of-words models. Thus, they are widely used in language modeling and some web applications. However, seldom does the approximation of the word-to-word relationship emerge; the words are independent from each other. In other words, the semantic information of the text is missing in the models. When dealing with high-dimensional vectors, the excessive dimensionality leads to an exponential expansion of the network computation and a high training-time cost.

The word embedding approach solves the shortcomings of the bag-of-words model by mapping each word to a low-dimensional vector of fixed length in space; then, the similarity between them can be calculated. Word2Vec is a classical word embedding model. There are two models for word2vec’s vocabulary training, namely the continuous bag-of-word model (CBOW) and the skip-gram [53]. CBOW predicts the word itself from the context in which it is found, and skip-gram predicts the word itself from the words that are likely to appear in its context. Both methods were lightly tested in the early stages of the work, and the latter model was eventually used because its word vectors allowed more adjustments in prediction than CBOW and were more likely to obtain better recognition results.

#### 6.1.2. Word Vector Optimization

The formation of a mature word vector expression can be measured by the following formulas:

(28) $$\log P\left(w_{o}|w_{c}\right)=u_{o}^{⊤}v_{c}-\log\left(\sum_{i\in \nu }\;\exp\left(u_{i}^{⊤}v_{c}\right)\right)$$

(29) $$\frac{\partial \log P\left(w_{o}|w_{c}\right)}{\partial v_{c}}=u_{o}-\sum_{j\in \nu }\;\left(\frac{\exp\left(u_{j}^{⊤}v_{c}\right)}{\sum_{i\in \nu }\;\exp\left(u_{i}^{⊤}v_{c}\right)}\right),$$

where $w_{o}$ and $w_{c}$ are the surrounding words and the specific center word, respectively. When the word $w_{c}$ represents the HT component, the corresponding $v_{c}$ is the word vector of the central word, $u_{i}$ is a word vector representing any word in the thesaurus, and $u_{o}$ represents the word vector for the surrounding component words. The circuit layout probabilities are calculated using the three word vector parameters described above. After probability normalization, $v_{c}$ learns enough path sentences to record the distribution of components around HT.

In order to make the word vector more accurately represent the circuit layout to identify HTs efficiently, Formula (29) [54] given in the form of partial derivatives is proposed, where $u_{j}$ makes the central word $w_{c}$ (the HT word) to be the word vector represented by the surrounding words. We minimize Formula (28) [54] in combination with the gradient descent algorithm and finally derive $v_{c}$ as the word vector needed. The above procedure has the same effect on learning the circuit layout when the words represent non-HT components.

#### 6.1.3. Setting the Related Parameters

The above pretrained word vector already contains the location information of the HT components in advance. This greatly reduces the effort of optimizing the model when further training the TextCNN. In this work, the word2Vec word vectors were trained with the skip-gram training mode, the sliding range size was set to five, and the word vector dimension was set to 100. More experimental parameters and analyses are described in the experimental section.

### 6.2. TextCNN Architecture and Parameter Details

A training model that fits the project is extremely important to the overall work. For TextCNN, in addition to the pretrained word vectors mentioned above, a reasonable neural network structure and a rich means of parameter optimization also influence the model’s detection of HTs. In this part, the TextCNN structure used in this paper and the setting of some parameters are introduced.

#### 6.2.1. TextCNN Construction

The core idea of the convolutional neural network (CNN) is to capture local features to achieve the prediction of the whole object. TextCNN applies a shallow CNN to text classification. The framework of TextCNN was constructed referring to [53], as shown in Figure 5.

All path sentences after text preprocessing were input to the input layer in order, and TextCNN used a one-dimensional convolutional layer to compute multiple convolutional kernel features for HT words and non-HT words in the sentences. The obtained results were put into the maximum pooling layer to select the most valuable features for identifying the HT. Then, the features were pooled in the fully connected layer. Eventually, multiple neurons were synthesized in the fully connected layer to obtain the judgment of HT/non-HT.

The word vectors were loaded with two methods: static and non-static forms. The static approach uses pretrained word vectors from the word2vec model and does not adjust the word vectors during each training session. The non-static approach uses pretrained word2vec vectors to initialize the word vectors and updates the word vectors in each iteration, which allows the model to converge faster. The actual experiment can be conducted in a static or non-static way depending on the environment, and the final results are acceptable. Moreover, the combination of static and dynamic word vector matrices can consider both the results of iterative non-updating and updating in TextCNN to optimize HT detection.

#### 6.2.2. Parameters’ Setting and Optimization

A. Sentence length setting: For the overhead of the model, the model of the global strategy considered the effect of the length of the sentence on the overhead. About 20% of the length was discarded, and the sentence length was set to 100. The local strategy was left untreated, since it has a controllable parameter δ.

B. Convolution kernel width setting: Both strategies proposed in this paper used three convolutional kernels of different widths, and the number of convolutional kernels used by the two strategies was different. This was obtained by testing lightly to preserve the meaning of the parameters to the maximum extent possible. The specific descriptions are mentioned in the experimental section.

C. Dropout setting: The dropout value in the fully connected layer was 0.5 by default to alleviate the overfitting in both the global and local strategies.

## 7. Experiments and Evaluation

In this section, several experiments are described that evaluated the MHTtext model proposed in this paper. Due to the differences in the dependencies on the two strategies in different environments, experimental parameters with adaptations were designed for both strategies. Then, the experiments were analyzed specifically for each of the strategies. In this work, the proposed HT recognition framework for TextCNN was implemented by PyTorch code framework in Python language and tested on personal computers (PCs), such as an i5-7400HQ/i5-10500H CPU, GTX1050Ti/2080Ti graphics card and 16 GB/32 GB RAM. The datasets used for the experiments in this paper were all from Trust-HUB [55,56,57]. The datasets were Verilog gate-level netlist files, and each file was labeled with normal and Trojan modules. The hardware Trojans may pose the threat of signal tampering.

Due to the limitations of the experimental dataset and to better illustrate the different focus of the two strategies, extreme values were used for some of the parameters described in Section 4. In the experiments for the global and local strategies, θ was set as $max\left\{L_{1}^{\tau },\;L_{2}^{\tau },\;\ldots ,\;L_{i}^{\tau }\right\}$ and 0, respectively, in the hope of simplifying the experiments. Subsequent work will consider more complex dynamic parameter scoring mechanisms.

### 7.1. Global Strategy Experiments

The number of words in the vocabulary in the pretraining stage was 15, which was the result of the de-duplication and cleaning of all the words in the netlist sentence.

**(1) Pre-stage of the global strategy**

**Figure 6:**
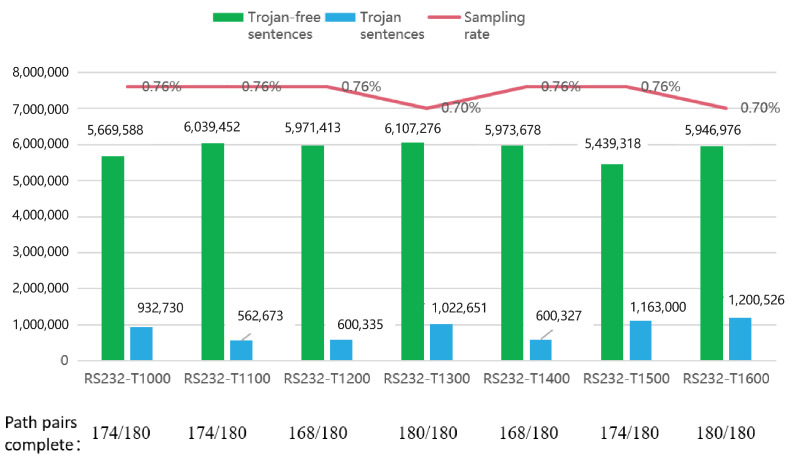
Complete extraction rate, label statistics, and sampling coverage in seven netlists.

In the netlist preprocessing step, each netlist had 180 path pairs. Figure 6 shows the number of non-timeout path pairs in each benchmark test netlist. According to the overall statistics, only a few path pairs were not fully extracted. This indicates that the generated sentences nearly expressed the topology of the circuit. Furthermore, the topological expressions of these time-out path pairs could be supplemented in other fully extracted path pairs because the non-timeout path pairs could pass through the blank topology behind the time-out path pairs in some situations.

**(2) Training detail of the global strategy**

In the training model stage of TextCNN, the training and testing sets were divided according to the leave-one-out approach. That is, the sentences generated from one netlist at a time were used as the testing set, and the sentences from the rest of the netlists were used as the training set. Since the number of sentences collected from the netlist was too large to obtain better experimental results, a balanced sampling of the dataset was performed. That is, 25,000 positive samples (with HT sentences) and 25,000 negative samples (with HT sentences) were randomly selected from each netlist, and all netlists formed a total of 50 such sampled datasets. The number of samples was determined as a result of the combination of the dataset and the computational overhead required for neural network training. According to Figure 6, the amount of data sampled in a single group (350,000 sentences in total) represented about 0.74% of all the data. On the other hand, the sentences generated in a single path pair were repetitive in terms of expressing the circuit structure. After all, if there were many-to-many or even many-to-many components of the same type in paths with the same starting and ending points, then the expressions of the content of this part of the sentence were necessarily the same.

For the TextCNN settings, since there was a one-dimensional convolution layer, only three types of widths to convolution kernels were selected, which were 3, 4, and 5, respectively. There were two convolution kernels of each width and a total of six kernels. Two groups of three kernels with different widths were assigned to the static word vector matrix and the dynamic word vector matrix. More clearly, the basic selection of several settings is shown in Table 3.

Although a single sampling can reflect the effect of the model, considering the influence of chance factors, the combined sampling can make the results more realistic to the model. Therefore, 50 random samples were selected for the experiments of the global strategy to form a multi-group model. Fifty sets of sampled training data were tested sequentially on an RS232-netlist, and a total of 350 training models was generated in the experiments.

### 7.2. Local Strategy Experiments

The experiments designed for the local feature strategy of the circuit used word2vec pretrained word vectors as the original input, and the word vector dimension was set to 100. Three/ten convolutional kernels of sizes 2, 3, and 4 were used. In the local strategy, the non-static word embedding pattern was used to compensate for the possible detection effect of an inadequate search scope selection in the early stage of the experiment, together with the controllable variable δ. The dropout parameter was set to 0.5 after the convolutional layer, the training batch size was 128, Adam was selected as the optimizer, and cross entropy was selected as the loss function. More clearly, the basic selection of several settings is shown in Table 3. The selection of the local strategy validation method used the tenfold cross-validation method. The mean data and the optimum data are presented in the experimental part of this paper for comparison.

For the selection of the search scope parameter δ, we also conducted a lightweight test in an attempt to achieve a balance between the time cost and effectiveness by rationalizing the selection of δ. This is the reason why the local-feature-based strategy was proposed in this paper. After a lightweight test, for which the data are shown in Table 4, δ was set to three as an empirical value. The time-cost result was acceptable in terms of the time budget of the whole model. Different environments may be suitable for different parameters. In the treatment of this issue, a readily acceptable vested parameter was used as a criterion in this paper, and all subsequent experiments were based on the criterion parameter for lightweight testing to determine its value.

### 7.3. Evaluation

The evaluation results of the experiments were derived from the machine learning indicators, i.e., the true positive rate, true negative rate, precision, accuracy, and the F-measure. The paper also uses a custom indicator SEI to quantify the detection data more efficiently.

#### 7.3.1. Previous and Traditional ML Indicators’ Evaluation

This part compares the experimental data of the two strategies and further compares a variety of models.

**(1) Model sample distribution**

**Figure 7:**
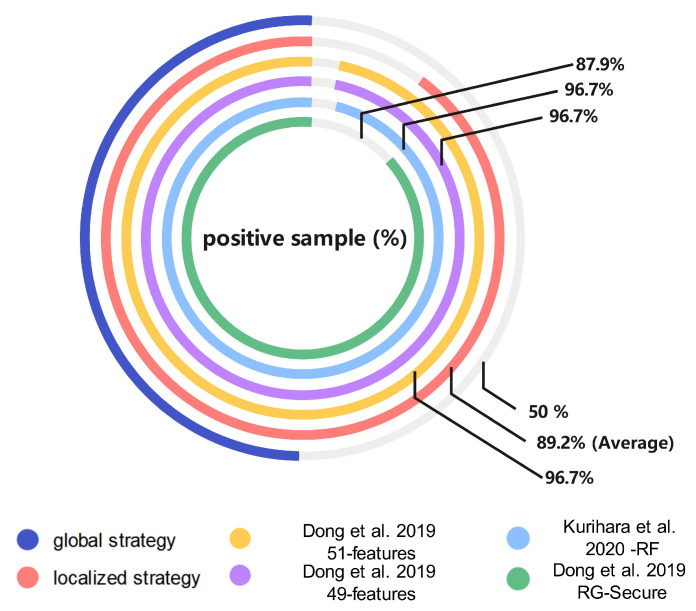
The sample distribution of the comparison models (Our - glo, Our - loc, Dong et al. 2019 - 49/51 features [38], Kurihara et al. 2020 - RF [30], Dong et al. 2019 RG-Secure [39]).

**Figure 8:**
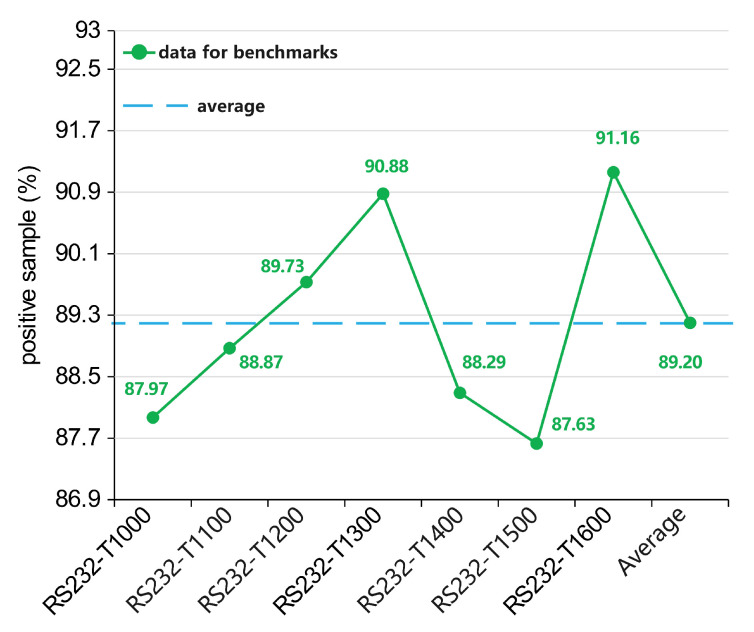
The sample distribution of the local strategy model.

Figure 7 shows the division of the netlist samples in several models. Positive samples (PS) and negative samples (NS), respectively, represent the HT samples and non-HT samples. The global strategy model relies on a balanced data set for training, which makes the structure of the dataset better than the other five models that depend on the wire to judge the HT. Note that the local strategy proposed in this paper did not perform balanced sampling, and the sample distribution was relatively unbalanced due to the limitations of the dataset and the aim of preserving the local characteristics of the individual parts of the circuit logic. The details of the local strategy sample data are presented in Figure 8, and the sample distribution remained stable within a certain range. Moreover, the PS and NS concepts in reference [58] were contrary to all the other comparison models; so, the paper recalculated them.

**(2) Feasibility and data comparison of the global and local strategies**

Table 5 and Table 6 list the HT detection effect of each netlist of the training models. These experimental results were sufficient to declare that the proposed two methods were feasible with incomplete data collection. At the same time, it can be seen that the global strategy had better performance in all cases than the DFS-based local strategy. The sentences extracted by the global strategy were long texts, which had more valid information. For the local strategy, the experiments also demonstrated that the local feature model with the controllable parameter δ could also achieve good results. Making the parameter δ adjust autonomously to the needs of the environment may maximize the advantages of the local strategy. The optimal and mean values of the local strategy in the experimental group are presented in Table 6. The data in the mean effect table show that the local strategy also achieved the desired effect by controlling the time cost.

**(3) Traditional machine learning indicators’ evaluation**

**Figure 9:**
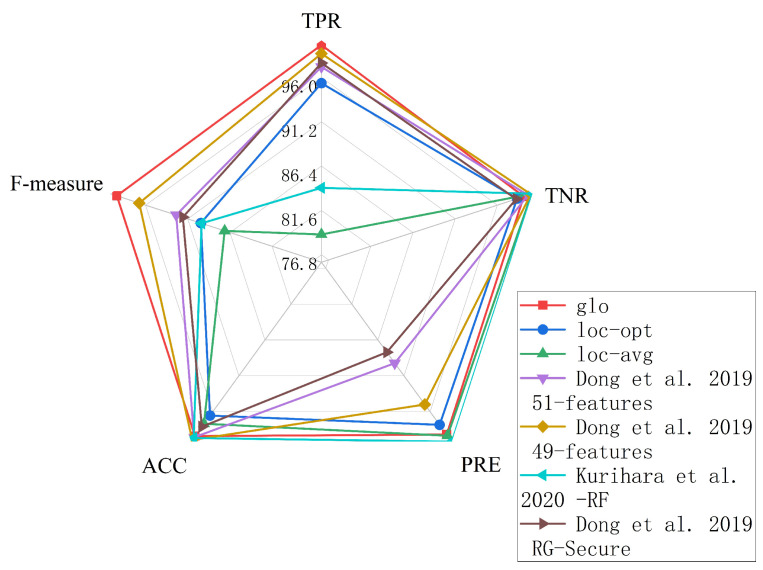
Comparison results for five ML indicators (Our - glo, Our - loc-opt, Our - loc-avg, Dong et al. 2019 - 49/51 features [38], Kurihara et al. 2020 - RF [30], Dong et al. 2019 RG-Secure [39]).

Figure 9 compares the performance of the centrally different methods on the machine learning indicators [30,38,39]. The results showed that the proposed global strategy model was the best on the TPR and F-measures and ranked second on the PRE. Although both the TNR and ACC were only fourth, they were only about one percentage point behind the leaders, which can be considered at the same level. The results of all the average ML indicators were higher than 99%. The fact proves that the global strategy model is feasible. Moreover, the overall performance for the other methods had large fluctuations compared with this work, and the local strategy also achieved the desired results while controlling the time costs with δ.

#### 7.3.2. Stabilization Efficiency Index (SEI) Evaluation

In order to fit the abstract problem itself presented in Section 4 of this paper, the experimental group introduced a new evaluation indicator, the stabilization efficiency index (SEI) after extensive calculations and analysis. With the help of the SEI, it is possible to quantify more effectively the stability of the model in detecting HTs. The SEI can be described as:

(30) $$SE_{HT}=\frac{avg(TPR)+avg(TNR)}{\sigma (TPR)+\sigma (TNR)+1},$$

where avg(TPR) and avg(TNR) represent the mean values of the TPR and TNR, respectively. σ(TPR) and σ(TNR) are the TPR standard deviation and TNR standard deviation, respectively. The standard deviation can be calculated by the following formula:

(31) $$\sigma =\sqrt{\frac{1}{N}\sum_{i=1}^{N}{\left(x_{i}-\mu \right)}^{2}},$$

where μ is the expectation. From Formulas (30) and (31), it can be obtained that if the model has a higher ability to correctly identify the PS and NS, and the performance fluctuation for each netlist is smaller, then it has better stability in general. In Formula (30), the denominator should be greater than 1 to circumvent the effects that arise in the case of less than 1 (the amplification effect of the ratio). Normally both the numerator and denominator have the same trend, as the model does not fluctuate significantly in stability and accuracy from one dataset to another. However, in a practical setting, uncertainties in the dataset and model parameter settings can cause several of the parts to have different effects from the others. This difference is magnified if compared to a more standard model. This is a reflection of the requirements of the SEI indicator in terms of the accuracy and stability of the model balance.

For more indicators, the following formula is defined:

(32) $$SE_{overall}=\frac{\sum_{i\in FI}avg\left(i\right)}{(\sum_{i\in FI}\sigma \left(i\right))+1},$$

where $FI=\left\{TPR,\;TNR,\;PRE,\;ACC,\;F-measure\right\}$.

Formula (32) is obtained by the further expansion of Formula (30). Several key indicators from traditional machine learning are considered together to obtain a more comprehensive measure.

**(1) Performance of each model on the SEI**

**Figure 10:**
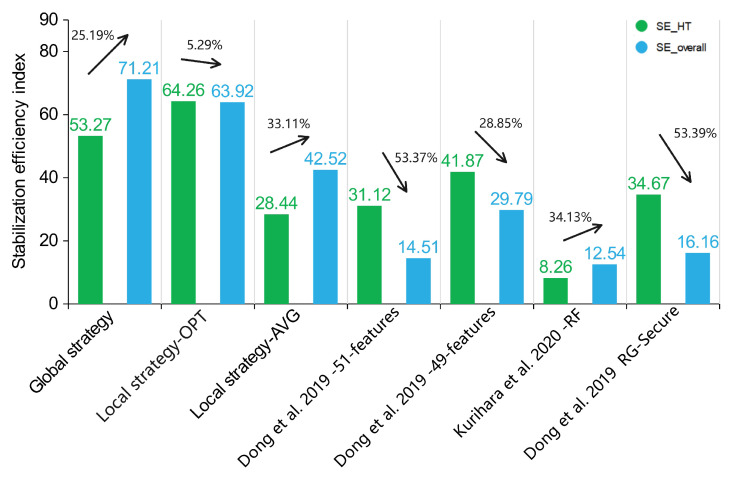
Statistical values of the $SE_{HT}$ and $SE_{overall}$ on each model (Our - glo, Our - loc-opt, Our - loc-avg, Dong et al. 2019 - 49/51 features [38], Kurihara et al. 2020 - RF [30], Dong et al. 2019 RG-Secure [39]).

In Figure 10, the global strategy model achieved high scores of 53.27 and 71.21 on the $SE_{HT}$ and $SE_{overall}$, respectively, maintaining an acceptable position. The local strategy model achieved high scores of 64.26 and 63.92 on the $SE_{HT}$ and $SE_{overall}$ (OPT), respectively. On average, the local strategy also achieved acceptable results with the controlled time cost and was better than the other methods except for the global strategy. The other four models had lower SEI evaluations due to large performance fluctuations. Since the models for other references detected the HT based on the wire features, not only were the rare HT samples extracted (Figure 6 also shows the low proportion of HT samples in these models), but their feature calculations only considered the local range for the circuit. They obtained a fluctuating performance when using traditional ML algorithms to train on unbalanced datasets.

**(2) Evaluation of the two strategies on the SEI**

The global strategy model’s end-to-end path sentence expression depends on the word vector to include the information for the global circuit under the feature calculation of the TextCNN. Moreover, using the simple path, it can be found that multiple path sentences are passing through the same HT component. Then, it magnifies the HT information to meet the balanced training for the dataset. These factors are the core reasons for the stable performance of the proposed model. Table 7 shows a comparison of the two strategies in the MHTtext model.

On the $SE_{HT}$, the local strategy (OPT) obtained a relatively excellent value. This is because the global strategy did not perform well on the RS232-T1000 and RS232-T1300 in the experiment, but the other datasets were close to 100%. Then, it affected the TNR value to some extent. In contrast, the local strategy proposed as a balance of multiple elements (although not as accurate as the global strategy with the current parameter settings) was instead closer to the data with less fluctuation because it took into account the local characteristics of each part of the circuit logic. On average, on the other hand, it was still the global strategy that prevailed. However, the local strategy proved its controllable parameters δ could provide new possibilities for balancing both the time cost and effectiveness.

## 8. Discussion

In recent years, it has been discovered from a spate of security incidents that the security threats posed by hardware should not be underestimated. Hardware Trojan protection is an important topic in hardware security. Traditional hardware Trojan detection methods are effective for low integration and complexity chips. Post-silicon methods are inadequate for VLSI and 3D ICs. More importantly, these methods require gold chips as a reference, which are difficult to obtain or do not exist for specific chips. With big data and computing power, machines can easily be trained to bring satisfactory results.

At this stage, many research teams have tried to introduce deep learning into the VLSI hardware Trojan detection task. However, deep learning mostly requires a high time cost. Therefore, balancing the detection effect and time cost is also a problem that needs to be solved. Our experimental results show that the introduction of deep learning for netlist-level hardware Trojan detection can achieve excellent results. For different scenarios (e.g., the precise detection of hardware Trojans or the fast detection of a large number of chips, etc.), our proposed dynamic tuning scheme can effectively select a suitable strategy according to the actual situation. Due to the limitation of the existing datasets, we used the RS232 series dataset available on Trust-Hub, which contains hardware Trojans with the effect of changing the original circuit function. Since this paper focuses on detecting netlist-level hardware Trojans, this series of gate-level netlist data was used.

As with the other methods, our approach achieved excellent results detecting hardware Trojans on gate-level netlists. Moreover, we proposed a more comprehensive scheme to deal with different scenarios. The accuracy-driven global strategy is suitable for tasks that require high detection accuracy. Hardware Trojan detection methods that use a global strategy have strength in high accuracy performance because the model uses a complete path sentence representation implemented end-to-end from the boundary ports of the entire circuit topology. Long sentences allow a complete record of how the signal travels through the component paths from input to output. Multiple global port-level path sentences generated by a single netlist can be integrated and provide a complete representation of the circuit topology.

The hardware Trojan detection method using the local strategy had a faster detection performance because of the lightweight work on the sentence generation method. A path sentence generation expression for the local component domain was used for sentence generation. It only applied short path sentences to record topological information in a limited range around each component, and the range size can be customized to make it flexible in lightweighting. This detection scheme applies to small netlists and can be extended to larger netlists to compensate for the limitations of accuracy-oriented detection schemes in netlist size. It can even be considered for fast and coarse inspection work.

It was difficult to obtain the netlist of the implanted chip during the experiment, and most of the existing work on netlist-level hardware Trojan detection was performed on the benchmark provided by Trust-Hub; so, the types of hardware Trojans detected were also limited by the data provided by it. Hardware Trojan implantation and hardware Trojan detection are mutual game processes, and more effective methods will emerge by optimizing hardware Trojan implantation technology.

## 9. Conclusions

The design and production of a chip require many steps and are performed by multiple manufacturers. It is easy to implant hardware Trojans in this process. In this paper, an MHTtext model consisting of two strategies was proposed and successfully demonstrated that generating sentences corresponding to circuit logic information can effectively detect HTs. These sentences are constructed from actual signal transmission processes within the circuit ports. The two strategies proposed in this paper are each suitable for use in different environments. The global strategy can achieve better accuracy in most cases, and the local strategy has better performance in flexibility and cost control by balancing the time overhead and detection effectiveness according to controllable parameters δ. The automatic single-feature detection mechanism provides the possibility of automatic feature learning. Both strategies have perfect performances in their specific situations.

## Figures and Tables

**Table 1:** Comparison of machine-learning-based approaches.

Ref.	Stage	Type	Level	Benchmark	Overview
[30]	pre-silicon	static detection approach	gate-level	Trust-hub RS-	The authors evaluated hardware Trojan detection methods using neural networks and random forests at gate-level intellectual property (IP) cores that contained more than 10,000 nets.
[38]	pre-silicon	static detection approach	gate-level	Trust-hub RS-	The authors proposed new Trojan-net features and then filtered 49 new effective feature sets using the scoring mechanism of eXtreme Gradient Boosting. Further, the authors trained and detected hardware Trojan classifiers respectively based on the new feature sets by eXtreme Gradient Boosting algorithm.
[29]	pre-silicon	static detection approach	gate-level	Trust-hub RS-	The authors proposed the model named ML-HTCL. It uses the multilayer BP neural network for the control-signal-type HTs and the one-class SVM for the information leakage HTs, respectively.
[39]	pre-silicon	static detection approach	gate-level, RTL	Trust-hub RS-	At the gate level of chip design, the authors proposed an algorithm named lightGBM. The algorithm can quickly process high-dimensional circuit feature information and effectively improve the detection efficiency of hardware Trojans.

**Table 2:** Symbols and notations.

Symbols	Definitions
δ	the predefined search scope for the local strategy
$T_{spath}/T_{lpath}$	the simple path sentence generated from two strategies
$T_{spathHT}$/$T_{lpathHT}$	the HT simple path sentence
$SE_{HT}$	the models’ SEI for sample recognition
$SE_{overall}$	the models’ SEI for all ML indicators
θ	the unacceptable length of the logic component definition part mentioned in Definition 3 (signal flow logic)
Θ	the length of the logic component definition part with a preset standard netlist dataset such as RS232-T1000.
$\xi_{i}$	the tuple to record each specific component and its in-wire/out-wire information
$X_{ii}$, $X_{io}$	the sets of in-wire and out-wire of component $n_{i}$
$X_{iport}$, $X_{oport}$	the input port wire set and output port wire set
SC, EC	the node sets of the start components and end components
$f_{s}$	the signal transfer function in the circuit
$f_{sp}$	the simple path function
$n_{f}$	the specific end component in P
E(θ), $E_{loc}\left(\theta \right)$, and $E_{glo}\left(\theta \right)$	the total effect, the effect of the local strategy, and the effect of the global strategy

**Table 3:** Settings used for the global and local strategies.

Setting	Glo-Value	Loc-Value
Word embedding model	Word2vec	Word2vec
Model for word2vec	skip-gram	skip-gram
Word embedding dimension	100	100
Convolution kernel size	(3, 4, 5)	(2, 3, 4)
Number of convolution kernels	3*2	3*3, 3*10
Optimizer	Adam	Adam

**Table 4:** Lightweight tests for different scopes.

Benchmark	Search Scope δ	Time Cost	ACC
RS232-T1000	2	0.72 s	97.52%
RS232-T1000	3	0.83 s	97.83%
RS232-T1000	4	1.11 s	97.36%
RS232-T1000	5	2.30 s	96.94%
RS232-T1000	6	6.98 s	97.57%
RS232-T1000	7	23.75 s	94.35%

**Table 5:** The result of TextCNN’s classification for path sentences (GLO).

Benchmark	TPR	TNR	PRE	ACC	F-Measure
RS232-T1000	99.97%	95.95%	96.11%	97.96%	98.00%
RS232-T1100	99.95%	100.00%	100.00%	99.98%	99.98%
RS232-T1200	99.98%	100.00%	100.00%	99.99%	99.99%
RS232-T1300	99.99%	97.11%	97.19%	98.55%	98.57%
RS232-T1400	99.98%	100.00%	100.00%	99.99%	99.99%
RS232-T1500	96.75%	100.00%	100.00%	98.37%	98.35%
RS232-T1600	99.98%	100.00%	100.00%	99.99%	99.99%
**Mean**	99.51%	99.01%	99.04%	99.26%	99.27%

**Table 6:** The result of TextCNN’s classification for path sentences (LOC).

	Local Strategy - 1 - OPT					Local Strategy - 2 - MEAN				
**Benchmark**	**TPR**	**TNR**	**PRE**	**ACC**	**F-Mesure**	**TPR**	**TNR**	**PRE**	**ACC**	**F-Mesure**
RS232-T1000	95.1%	98.1%	98.5%	96.4%	91%	84%	99.90%	99.4%	98.0%	91%
RS232-T1100	95.2%	97.1%	97.8%	96.2%	85%	81.90%	99.90%	99.50%	97.9%	90%
RS232-T1200	96.7%	98.9%	98.9%	97.1%	95%	72.50%	99.90%	99.2%	97.1%	84%
RS232-T1300	93.2%	99.5%	98.1%	95.8%	93%	82.40%	99.90%	99.30%	98.3%	90%
RS232-T1400	96.4%	97.3%	97.9%	96.8%	88%	85.70%	99.90%	99.40%	98.3%	92%
RS232-T1500	94.3%	98.5%	96.9%	95.5%	84%	73.30%	99.90%	98.7%	95.5%	84%
RS232-T1600	96.7%	98.4%	96.1%	97.8%	92%	73.40%	99.9%	98.90%	97.8%	84%
**Mean**	95.37%	98.26%	97.74%	96.51%	89.71%	79%	99.90%	99.20%	97.6%	87%

**Table 7:** Comparison of the two strategies on the SEI ($SE_{HT}$ and $HT_{overall}$).

	TYPE	TPR	TNR	PRE	ACC	F-Measure	${\mathrm{SE}}_{\mathrm{HT}}$	${\mathrm{SE}}_{\mathrm{overall}}$
1	AVG(%)	99.51	99.01	99.04	99.26	99.26	**53.27**	**71.21**
	σ	1.13	1.60	1.54	0.85	0.85		
2	AVG(%)	95.37	98.26	97.74	96.51	89.71	**64.26**	**63.92**
	σ	1.23	0.79	0.88	0.73	3.8		
3	AVG(%)	79.0	99.9	99.2	97.6	87	**28.44**	**42.52**
	σ	5.29	0.00	0.27	0.92	3.40		

1 GLO, 2 LOC-1-OPT, 3 LOC-2-MEAN.

## Data Availability

The data presented in this study are available on request from the corresponding author.
